# Homologous Recombination Deficiency Testing for BRCA-Like Tumors: The Road to Clinical Validation

**DOI:** 10.3390/cancers13051004

**Published:** 2021-02-28

**Authors:** Marjolijn M. Ladan, Dik C. van Gent, Agnes Jager

**Affiliations:** 1 Department of Molecular Genetics, Erasmus MC Cancer Institute, Erasmus University Medical Center, 3000 CA Rotterdam, The Netherlands; m.ladan@erasmusmc.nl; 2 Oncode Institute, Erasmus University Medical Center, 3000 CA Rotterdam, The Netherlands; 3 Department of Medical Oncology, Erasmus MC Cancer Institute, Erasmus University Medical Center, 3000 CA Rotterdam, The Netherlands; a.jager@erasmusmc.nl

**Keywords:** HRD, BRCAness, DNA repair, PARP inhibitors, DNA double strand breaks

## Abstract

**Simple Summary:**

Optimal cancer treatment requires the selection of patients based on the characteristics of their tumors. This review explores various strategies to select patients for DNA double strand break-inducing agents, such as PARP inhibitors. Patients with germline *BRCA1/2* mutations benefit from these inhibitors. However, patients with a functional defect in the homologous recombination repair pathway without germline *BRCA1/2* mutations also show a similar reaction to this treatment. The challenge is to identify the patients with BRCA-like tumors. Here, we review various strategies to select this group of patients and summarize the clinical evidence for their performance.

**Abstract:**

Germline *BRCA* mutations result in homologous recombination deficiency (HRD) in hereditary breast and ovarian cancer, as well as several types of sporadic tumors. The HRD phenotype makes these tumors sensitive to DNA double strand break-inducing agents, including poly-(ADP-ribose)-polymerase (PARP) inhibitors. Interestingly, a subgroup of cancers without a *BRCA* mutation also shows an HRD phenotype. Various methods for selecting patients with HRD tumors beyond *BRCA*-mutations have been explored. These methods are mainly based on DNA sequencing or functional characteristics of the tumor. We here discuss the various tests and the status of their clinical validation.

## 1. Homologous Recombination Deficiency and Cancer

The discovery of new biomarkers of tumor treatment response is the focus of many studies. Several markers have made it to the clinic, such as hormone receptor status for breast and prostate cancer. However, most anti-cancer drugs are prescribed based on overall efficacy in particular tumor types without preselection based on predictive biomarkers. Developing biomarkers to select the best treatment for each patient should increase patient prognosis and decrease unnecessary side effects and costs.

In this review, we describe methods to select patients who might benefit specifically from a specific class of DNA damaging agents that induce double strand DNA breaks (DSB), including platinum salts and poly-(ADP-Ribose)-polymerase inhibitors (PARPi). These DSB require homologous recombination (HR) for their repair [1,2]. Patients with a germline mutation in the breast cancer susceptibility genes *Breast cancer 1* (*BRCA1*) or *Breast cancer 2* (*BRCA2*) have a high risk of developing breast cancer or ovarian cancer [3] and show high sensitivity to these DNA damaging agents [4,5]. As both BRCA1 and BRCA2 proteins are involved in HR [6,7], tumors without *BRCA* gene mutations but with a similar homologous recombination deficiency (HRD) phenotype are expected to show similar responses to DSB-inducing drugs. Currently, germline *BRCA* (*gBRCA*) mutated ovarian and breast cancer patients are eligible for PARPi treatment, but a clinically validated test to select HRD tumors (also called BRCA-like tumors) is not yet available.

**Figure 1:**
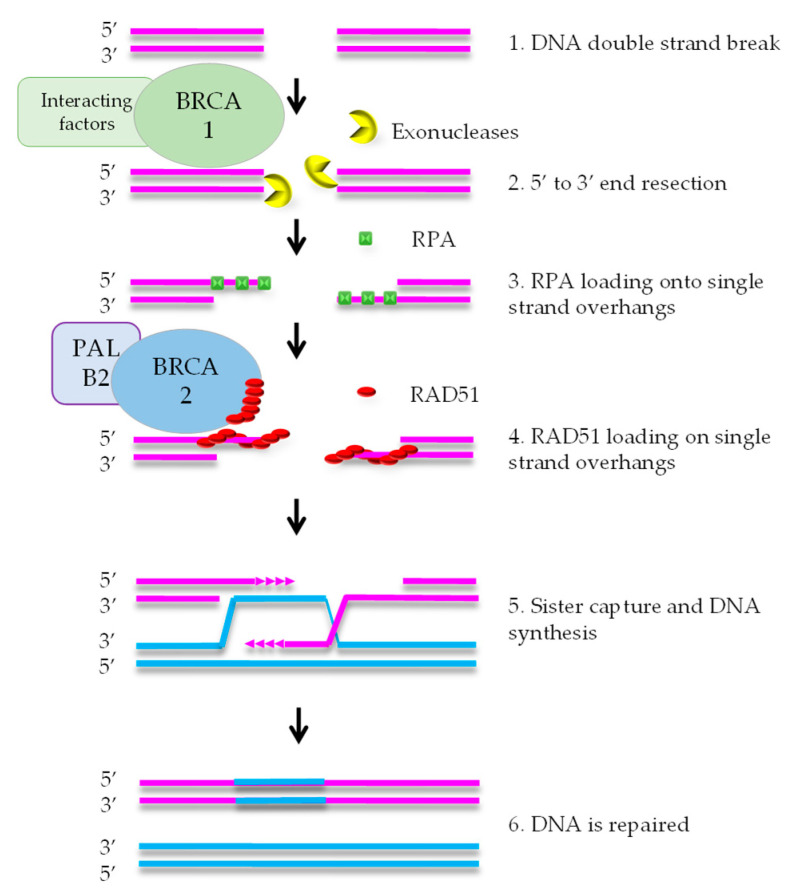
Simplified scheme of the homologous recombination pathway. After attraction of BRCA1 to the DNA ends, it stimulates exonucleases to create single-strand overhangs by 5′-3′ end resection. RPA binds to this single strand DNA. Subsequently, RAD51 replaces RPA, a reaction that also requires BRCA2 and PALB2. This DNA-RAD51 filament searches for homology in the sister chromatid that is subsequently used as template for DNA repair.

HR mediates error-free repair of DSBs that arise during DNA replication [8]. BRCA1 is attracted to DNA ends, where it stimulates exonucleases that create a single strand (ss) overhang by 5′-3′ end resection (Figure 1). RPA binds to the ssDNA and is subsequently replaced by RAD51 protein. This exchange reaction requires a complex containing RAD51, BRCA2, and PALB2. After formation of the RAD51-DNA filament, the single strand overhang searches for homology in the sister chromatid, that functions as the template for DNA repair. As HR uses the newly formed sister chromatid as a template, it is restricted to the S and G2 phases of the cell cycle [7].

DNA repair defects have been described as ‘enabling characteristics of cancer’ [9]. This means that defects such as HRD promote tumorigenesis by increasing the speed of mutation accumulation in a cell on its way to become a cancer cell. The DNA repair defect is often an early event in tumor evolution and the defect is present in the large majority, if not all, of the cells in the tumor. Therefore, HRD is a promising therapeutic target.

## 2. Various Ways to Define HRD

In the search for BRCA-like tumors, several tests have been designed to recognize them. Most tests are based on sequencing of the tumor compared to germline DNA. The most obvious test is sequencing of the known disease-causing genes, i.e., *BRCA1* and *BRCA2*. As an extension of this approach, panels of multiple genes can be chosen based on their involvement in the HR pathway, such as *PALB2* or *RAD51* (Figure 1; see Section 3 for examples).

Sequencing can also be used in the search for so-called genomic scars. These are specific types of genomic changes that are a direct or indirect consequence of the DNA repair defect, which accumulate over the course of tumor evolution (examples in Section 5).

Alternative tests are based on the loss and amplification of pieces of chromosomes or whole chromosomes. The basic idea behind these tests is that HRD tumors are often accompanied by very specific chromosomal alterations that confer a selective advantage in combination with *BRCA* gene mutations and the HRD phenotype, which can therefore be used as a way to identify BRCA-like tumors. These chromosomal gains and losses lead to changes in gene expression, which presents another possible way of identifying HRD tumors (examples in Section 4, Section 6, and Section 7).

Section 8 covers functional assays, which present a completely different approach. These assays directly investigate functional parameters in fresh tumor specimens. In general, a fresh tumor part receives a relevant treatment ex vivo and the response is measured to assess whether the tumor is HRD and thus BRCA-like.

**Figure 2:**
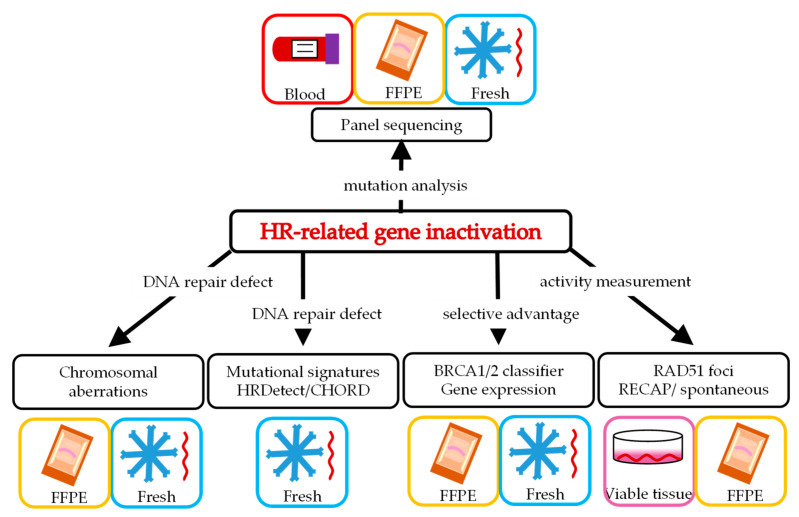
Homologous recombination deficiency (HRD) biomarkers and material needed to analyze the particular biomarker (see text for details). FFPE = formalin fixated paraffin embedded; Fresh = fresh frozen material; HR= homologous recombination.

The various ways of selecting patients require different starting material (Figure 2) and are described in more detail below. In addition to the analytical validity of such potential biomarkers for BRCA-like tumors, we describe the available clinical validation of the individual HRD tests.

## 3. Panel Sequencing of Potential Causal Genetic Changes in HR Genes

HRD can not only be caused by *BRCA1* or *BRCA2* gene mutations, but also by mutations in other HR genes. Therefore, sequencing of a panel of candidate HR genes is a possible method to select BRCA-like tumors. Mutations in the *PALB2* gene are a known cause of hereditary breast cancer and ovarian cancer, and have been found in 0.1–2.7% of unselected Breast Cancer (BrC) patients [10]. Other genes have been selected based on their known role in the HR pathway, such as *RAD51* or *RAD54.* The selection of HR associated genes can differ between panels.

As a first evaluation of this approach, a panel of 102 HR pathway related genes was analyzed across various cancer types using data available from The Cancer Genome Atlas (TCGA). Bi-allelic mutations in HR-related genes were compared to features of HRD deduced from alterations throughout the genome [11]. The authors found significant correlation between biallelic pathological gene alterations and mutation patterns which have also been shown to be associated with mutations in *BRCA1/2* genes. Moreover, in a cohort of ovarian cancer patients treated with carboplatin, the biallelic alterations were clearly associated with a longer overall survival, suggesting that the gene panel approach indeed selects *BRCA*-like tumors (also referred to as BRCAness).

Further clinical evidence for the predictive value of the HRD test based on mutations in HR genes came from studies in patients with castration-refractory prostate cancer (CRPC; Table 1). In a single-arm phase 2 study, a mutation in one of the 113 non-*BRCA* HR-related genes in tumor tissue of CRPC patients was associated with a high combined response rate (CCR) to olaparib (7/9 = 78%) [12]. In the larger TOPARP-B phase 2 study, the overall CCR among CRPC patients with HR-related gene mutations was lower (20/66 = 30.3%) [13]. Analysis of subgroups with mutations in single HR genes showed higher CCR in some (*ATM* or *PALB2* gene mutations), but lower CCR in other subgroups (e.g., *CDK12* gene mutations). Recently, the results of the randomized phase 3 PROfound study in patients with CRPC, comparing the effectiveness of olaparib with enzalutamide or abiraterone, were published [14]. Tumors with mutations in at least one out of 15 HR-related genes were expected to benefit more from olaparib than from enzalutamide or abiraterone. Although the primary endpoint, imaging-based progression-free survival (iPFS), was higher for olaparib than enzalutamide in patients with a *BRCA1/2* or an *ATM* mutation (iPFS of 7.4 versus 3.6 months; HR 0.34 (0.25–0.47)), this was not the case in patients with mutations in other HR-related genes (estimated iPFS of around 2.8 vs. 3.2 months). In addition, subgroup analysis in patients with only an *ATM* mutation showed no preferential benefit of olaparib treatment compared to enzalutamide or abiraterone. This suggests that the longer iPFS on olaparib in patients with HR-related gene mutations compared to enzalutamide or arbiraterone is mainly determined by the tumors with a *BRCA1/2* mutation, rather than tumors with mutations in non-*BRCA* HR-related genes. Based on these studies in CRPC patients, it is uncertain whether tumors with HR-related mutations other than *BRCA1/2* are truly HRD. Further investigation is required to determine which non-BRCA CRPC patient will benefit of PARPi treatment.

HR-related gene panels as a biomarkers for HRD have also been investigated in breast cancer patients, with somewhat conflicting results (Table 1). First-line gemcitabine/cisplatin (GP) treated metastatic triple negative breast cancer patients showed longer PFS than gemcitabine/paclitaxel (GT) treated patients in the subgroup with proven germline HR-related mutations in the randomized phase 3 CBCSG006 study (PFS 10.4 vs. 4.3 months respectively, *p* = 0.011) [15]. Even after excluding patients with a germline *BRCA1/2* mutation, this difference remained (estimated PFS of 10.9 vs. 4.7 months). In line with these findings, Tung et al. showed in the small TBCRC 048 study among patients with pre-treated metastatic breast cancer that 33% (9/27) of the patients with a germline HR-related gene mutation showed an objective response to olaparib monotherapy [16]. Remarkably, all patients with a response turned out to be patients with a germline *PALB2* mutation. However, somatic HR-related gene mutations, even somatic *PALB2* or *BRCA1/2* mutations, did not have predictive value for a response to olaparib (objective response rate 0% (0/10)) in this pretreated patient population. This may be different for treatment-naïve tumors given the findings of the PETREMAC study [17]. In this small neoadjuvant study among 32 triple negative breast cancer patients, somatic mutations in non-*BRCA1/2* HR-related genes were found to be predictive for a response to olaparib (objective response rate 100% (5/5)).

Finally, HR-related gene panels were examined in two studies among patients with ovarian cancer (Table 1) [18,19]. In both studies, the presence of somatic HR-related gene mutations other than *BRCA1/2* mutations had predictive value. In the prospective cohort study in which patients with primary ovarian cancer were treated with adjuvant carboplatin/paclitaxel, platinum sensitivity was found to be greater among tumors with an HR-related gene mutation compared to those without such a mutation [19]. Even in patients with extensively pretreated ovarian carcinoma, olaparib was found to be of added value over placebo in patients with somatic HR-related gene mutation [18].

In conclusion, the gene panel approach appears to have benefits for identifying BRCA-like tumors, although more research is needed to determine which genes should be included in this analysis.

## 4. Chromosomal Aberrations as a Consequence of HRD

HR is required to maintain chromosomal integrity by the precise repair of DSBs. Defects in this repair pathway result in imprecise repair and loss of chromosomal sequences, also referred to as loss of heterozygosity (LOH), as well as structural rearrangements (such as translocations). The level of chromosomal aberrations has therefore been suggested to correlate with HRD status.

Attempts to preselect HRD tumors based on the level of LOH throughout the genome has been described in the ARIEL2 (phase 2; single arm [20]) and ARIEL3 (phase 3; placebo-controlled [21]) studies, that examined the effect of the PARPi rucaparib in platinum-sensitive ovarian cancer (Table 2). Within the studies, three subgroups were defined: the *BRCA* mutated group, the non-*BRCA* mutated LOH-high group (HRD defined as LOH >14% (ARIEL 2) or LOH >16% (ARIEL 3)) and the non-*BRCA* mutated LOH-low group. In the ARIEL3 trial, the effect of rucaparib was clearly greatest in the *BRCA*1/2 mutant group (16.6 months compared to 5.4 months in the placebo group). Although there was a statistically significant added value of rucaparib in the other two groups compared to placebo, the absolute median PFS increase was smaller than in the *BRCA* mutated group (Table 2). This suggests that the discriminating power of the LOH testing for selecting HRD tumors in ovarian cancer patients is suboptimal, as all groups benefited significantly compared to placebo. This is in line with the results of the small phase 2 PrECOG study of triple negative breast cancer patients. Tumors with high LOH (*n* = 50) showed a 66% chance of a good tumor reduction (residual cancer burden [RCB] 0/1) in response to neoadjuvant chemotherapy consisting of carboplatin, gemcitabine, and iniparib. However, one in five women with low LOH tumors (*n* = 15) also showed a RCB0/1 response after neoadjuvant chemotherapy [22].

The high percentage of LOH appears to be associated with a higher likelihood of response to DSB-inducing agents. However, it is not distinctive enough to withhold these drugs from patients with low LOH tumors. Therefore, additional chromosomal aberrations have been added to the level of LOH to identify HRD tumors. Deletion of stretches larger than 15 Mb but smaller than the whole chromosome [34], telomeric allelic imbalance (TAI), and large-scale transitions (LST) were found more often in *BRCA1/2* associated tumors [34,35]. TAI indicates that the copy numbers of the paternal and maternal alleles are not equal in the sequences close to the telomere. LST denotes a group of chromosomal aberrations involving large chromosomal regions resulting from DSBs more than 10 Mb apart [35]. The HRD score can then be calculated based on LOH, TAI, and LST. The lower limit of this test to select HRD was determined based on the lowest score found in proven *BRCA1/2* associated tumors. In a retrospective study of chemotherapy, naive breast cancer (*n* = 497) and ovarian cancer (*n* = 561) patients with known BRCA1/2 status were used to set the cut-off value at ≥42 [36]. Thus, all non-*BRCA1/2* mutated tumors that scored ≥42 on this test were considered to be HRD tumors. A similar HRD test is currently commercially available from Myriad, referred to as the MyChoice assay.

A lot of clinical data have been collected with this commercial MyChoice assay, especially among patients with ovarian cancer who were treated with PARPi (Table 2). From these studies, a number of things can be concluded. First, there is a clear prognostic value of the MyChoice assay. Ovarian cancer patients with a germline (blood) or somatic (tumor) *BRCA1/2* gene mutation had the best prognosis compared to non-*BRCA1/2* tumors. Furthermore, tumors without *BRCA1/2* mutations with a high HRD score had a more favorable prognosis than those tumors with a low HRD score (Table 2). There is a similar trend with regard to the predictive value of the MyChoice assay. Here again, the presence of a *BRCA* mutation had the strongest predictive value for selecting tumors sensitive to a PARPi. This was followed in descending strength by an high HRD score and finally an low HRD score. In other words, non-BRCA1/2 tumors with a high HRD score had an increased chance of PARPi sensitivity. On the other hand, having a low HRD score in a non-BRCA1/2 tumor was not selective enough to completely omit a PARPi in this group, because there was still demonstrable added value compared to placebo treatment. This also applied to the non-commercial variant of this HRD assay used in a pre-specified subgroup analysis of the large SCOTROC4, phase III study among untreated stage III/IV epithelial ovarian, fallopian tube, or primary peritoneal cancers [25]. Therefore, there is currently no clear added value for determining this type of HRD test to select patients who do or do not benefit from a PARPi in ovarian cancer patients.

In view of the broad effectiveness of PARPi in ovarian cancer patients, it might be better to look for a biomarker that selects those patients that do not benefit from this treatment than the current approach that tries to identify the group that does benefit. An attempt has been made to do so by lowering the value of the score that determines the classification of HRD high and HRD low. Although Hodgson et al. [18] were the first to show some evidence that lowering this value from ≥42 (standard) to ≥33 for defining the HRD-high group was indeed possibly more distinctive, this was not confirmed in the large randomized phase 3 VELIA study [23], and the pre-specified subgroup analysis of the SCOTROC 4 study [25].

The MyChoice assay has also been investigated among breast cancer patients in several studies [29,30,31,32,33] (Table 2). The prognostic value of the MyChoice assay was clearly demonstrated in a post hoc subgroup analysis of the large randomized SWOG S9313 phase 3 study, in which concomitantly administrated adjuvant doxorubicin with high dose cyclophosphamide was compared with similar drugs sequentially administrated [33]. The disease-free survival (DFS) was better for the 67% triple negative breast cancer patients with an HRD phenotype compared to non-HRD (10-year DFS: 70.5 vs. 58.3% respectively), and also when patients with *sBRCA1/2* mutation were excluded (10-year DFS: 74.4 vs. 58.3%, respectively). Unfortunately, the predictive value of the HRD test could not be studied, as both treatment arms underwent an equal total dose of double stranded DNA break-inducing agents. In the neoadjuvant Geparsixto study comparing the triplet paclitaxel, pegylated doxorubicin, and bevacizumab with the triplet plus carboplatin, an HRD phenotype based on the MyChoice assay more likely resulted in a pathological complete remission (pCR) when carboplatin was added to the triplet [30]. However, the interaction between therapy and HR status was not statistically significant. In two other studies comparing monotherapy taxanes to monotherapy platinum-containing therapy [29,31], the MyChoice assay did not show predictive value. In the fourth study [32], there was no platinum-free treatment arm, and therefore the predictive value of the assay could not be properly investigated.

Thus, in both high-grade serous or endometrial ovarian cancer and triple negative breast cancer, the predictive value of the MyChoice assay appears to be limited and should in any case not be used in routine clinical practice for the selection of HRD tumors.

## 5. Genomic Scars Resulting from HRD

Genomic alterations can be the result of the HRD phenotype. Imprecise repair after DNA damage due to a dysfunctional HR pathway leads to mutations. These genomic alterations can be the direct consequence of the DNA repair defect in the tumor, or they may have been caused by up- or downregulation of other DNA repair pathways as compensation for the original DNA repair defect. These alterations can be used as biomarkers for the HRD phenotype. Whole genome sequencing (WGS) data are used to detect the precise changes in nucleotide sequence that have accumulated in the tumor DNA during the course of tumor evolution. This combination of various alterations is collectively referred to as ‘genomic scars’ [37].

The pattern of these genomic scars was analyzed using an unbiased computational approach. The first sign that scars could be used to identify causes of DNA alterations and possible underlying DNA repair defects came from a systematic analysis of point mutations [38]. Each nucleotide substitution was classified based on the change itself and the nucleotides preceding and following the point mutation. Subsequently, 21 patterns of substitution mutations were characterized, also known as ‘mutational signatures’. Later on, more patterns have been added to this original set and some patterns have been linked to specific DNA damaging agents or DNA repair defects [39]. Signature 3 was specifically linked to *BRCA1/2* deficiency. A similar enrichment for signature 3 was found for *PALB2* and *RAD51C* mutations or epigenetic silencing of the *BRCA1* or *RAD51C* promoter, but not for inactivation of other cancer-associated genes, such as *CHEK2* and *ATM* [40]. In all cases, this association with signature 3 could not be used to select individual HRD tumors, because its prevalence was quite variable. Some tumors containing *BRCA* mutations did not show a high contribution of signature 3, while several other (probably non-HRD) tumors showed a high contribution of this signature [40].

Another important characteristic of *BRCA* deficient tumors, in addition to signature 3, is a pattern of deletions with short stretches of sequence homology (also termed ‘microhomology’) at the junctions [41]. Subsequently, more features of DNA sequence changes have been characterized, including DNA rearrangements and deletions [42]. These characteristics have been combined in the computational algorithm HRDetect that was originally trained on breast cancer WGS [43]. It integrates the values for these characteristics, where scores above 0.7 indicate HRD. Using this cut off, almost 99% of the bi-allelic *BRCA1/2* mutations in the training set were detected by the algorithm.

The first evidence for clinical validation of the HRDetect assay came from nine patients with triple negative breast cancer who underwent biopsies prior to neoadjuvant chemotherapy [43]. Five tumors with a high HRDetect score showed a pathologically complete remission in response to epirubicine-containing chemotherapy, a topoisomerase 2 inhibitor that leads to, among other things, double-stranded DNA breaks. The other four tumors with a low HRDetect score all showed residual disease after neoadjuvant chemotherapy. In the ongoing population-based observational SCAN-B study, the prognostic value of the HRDetect assay was subsequently demonstrated in 145 triple negative breast cancer patients [44]. For example, multivariable analysis showed a statistically significant and clinically relevant 69% lower risk of distant relapse-free survival in patients with a HRDetect high compared to HRDetect low score (HR 0.31 (0.13–0.76)). However, a convincing predictive value for preferential benefit of anthracycline-containing therapy could not yet be demonstrated in the HRDetect high (62%) compared to the HRDetect low score tumors (38%). This may partly be the result of the relatively short median follow-up duration (<5 years) and the retrospective nature of this study.

The HRDetect assay was also investigated in the exploratory neoadjuvant RIO study in which 43 patients with triple negative breast cancer were treated with the PARPi rucaparib for two weeks prior to neoadjuvant chemotherapy or resection [45]. As in the SCAN-B study, the majority (69%) of the tumors showed a high HRDetect score, suggestive of a HRD phenotype. In addition, patients with a high score HRDetect tumor had a lower tumor burden (measured as lower levels of ctDNA) after 2 weeks of treatment with the PARPi rucaparib than patients with a low HRDetect score. However, there was no difference in the probability of a Ki67 reduction <50%, the primary endpoint of the study, between the tumors with an HRDetect high or an HRDetect low score. Further research is necessary to determine the predictive value of HRDetect for sensitivity to double strand DNA break inducers.

A similar test based on genomic scars is the Classifier of Homologous Recombination Deficiency (CHORD). This algorithm was developed using more than 3000 tumor WGS sets covering 31 types of cancer. Deletions with microhomology at the junction were most predictive for the HRD probability. The majority of tumors marked as HRD by the CHORD test were caused by biallelic inactivation of *BRCA1, BRCA2, RAD51C* or *PALB2*. However, 85 out of 211 identified HRD tumors did not harbor any of these mutations [46]. This test appears to be roughly equivalent to HRDetect, although currently no data have been published regarding the predictive value of the CHORD test in clinical practice.

## 6. The BRCA-Like Classifier as Predictor for HRD

Loss of function mutations in the *BRCA1* and *BRCA2* genes not only increase the chance of developing cancer, but they also decrease the viability and proliferative capacity of normal cells. Mutations in other genes are required to compensate for this selective disadvantage (for example inactivation of the *TP53* gene [47]). In other words, HRD can cause selective pressure towards a loss or gain of genes and (parts of) chromosomes. Therefore, specific chromosomal gains and losses have been analyzed to obtain evidence for HRD in tumors.

Initially, Linn and colleagues developed a *BRCA1*-like classifier for the selection of breast tumors with a HRD phenotype using a comparative genomic hybridization (CGH) assay [48]. In a retrospective analysis of a randomized phase III trial among stage III HER2 negative breast cancer patients, this *BRCA1*-like classifier proved to be a strong predictive factor for better outcomes after treatment with high-dose alkylating chemotherapy with autologous stem cell transplantation compared to standard anthracycline-containing therapy (risk of recurrence in BRCA1-like^CGH^ HR 0.12 (0.04–0.43) vs. non-*BRCA1*-like^CGH^ HR 0.78 (0.50–1.20); *p* interaction 0.006) [49]. The incidence of HRD tumors based on the *BRCA1* like classifier in this study was 33%, among the HER2 negative breast cancers. Among the triple negative breast cancer patients, this was much higher (57%), which is comparable to the percentages of HRD among triple negative tumors demonstrated by other HRD tests, such as the MyChoice assay. Extension of the BRCA classifier with a *BRCA2*-like CGH assay showed a higher percentage of HRD tumors [50]. This was, however, almost entirely explained by a HRD phenotype detected among ER-positive tumors (3% HRD based on the *BRCA1*-like^CGH^ classifier and 22% HRD tumors based on the *BRCA1/2*-like classifier). In view of the much higher percentage of HRD tumors among triple negative breast cancer, the *BRCA1*-like^CGH^ classifier was further investigated in this group. The predictive value of this classifier was confirmed in a retrospective case control study where the BRCA1-like^CGH^ tumors benefited from high-dose alkylating chemotherapy (overall survival in *BRCA1*-like^CGH^ HR 0.15 (0.03–0.83) vs. non-*BRCA1*-like^CGH^ HR. 0.93 (0.52–1.64); *p* interaction 0.045)) [51]. In a third retrospective study, however, the predictive value of the *BRCA1*-like^CGH^ classifier could not be confirmed [52]. In the ongoing prospective randomized phase 3 SUBITO study (NCT02810743), currently running in the Netherlands and France, the definitive predictive value of the *BRCA1*-like^CGH^ classifier will be determined.

## 7. Gene Expression Profiles as Predictor for HRD

Changes in chromosomal content will also affect gene expression levels and thus can be used to identify HRD tumors. On the basis of publicly available microarray datasets of 61 proven ovarian carcinomas, of which 34 have a proven germline *BRCA* mutation, a 60 gene profile was established that distinguished *BRCA* from non-*BRCA* tumors [53]. This gene profile was then tested in 70 ovarian cancer patients treated primarily with platinum-based chemotherapy and resection. As with other HRD tests, this profile showed a favorable prognosis of those tumors classified as HRD compared to non-HRD. However, no information on the predictive value could be given, as all patients underwent platinum-based therapy. A gene expression profile was also established in breast cancer patients, distinguishing *BRCA*-like (based on copy number variations) and non-*BRCA*-like breast tumors [54]. Of the 116 Her2 negative primary breast cancer patients, 47% showed a gene expression profile consistent with HRD (78% among triple negative and 14% among the ER+, Her2− breast tumors). This gene expression profile seemed predictive of a favorable response to the addition of the PARPi veliparib and carboplatin to paclitaxel compared to paclitaxel alone; however, this effect could only be demonstrated in the ER+, Her2− breast tumors. Furthermore, both gene expression profile studies were performed on tumors that had not previously been treated with chemotherapy. The question is what the predictive value of such gene expression profiles will be in larger studies, as well as the value of such profiles when they are determined on tumor material that has already undergone chemotherapy treatment.

## 8. Functional Tests

Intuitively, a functional test should be the ultimate HRD assay. The most straightforward form of functional testing is drug sensitivity screening of cell lines, organoids or tissue slice [55]. Although such tests may be able to predict patient outcome to therapy, they can be time consuming and they do not report on the HRD status of the tumor. Therefore, alternative functional assays have been developed for HRD selection. Most functional biomarkers in this respect are based on measuring RAD51 accumulation on DSB, generally referred to as RAD51 foci. RAD51 is involved in the HR pathway downstream from the BRCA1/2 proteins (see Figure 1). The RAD51 protein that covers the single-stranded DNA at double strand breaks can be visualized as RAD51 foci after immunofluorescent staining (Figure 1 step 4). These foci are absent in *BRCA1/2* deficient tumors, since RAD51 is not loaded onto single-stranded DNA ends in the absence of *BRCA1/2* [56].

### 8.1. HR Pathway Proteins after In Vivo Treatment

As a first approach, HR pathway protein accumulation could be determined on tumor biopsies collected before and after patients had received neoadjuvant treatment with DSB-inducing chemotherapy. In the first study, biopsies of 68 breast cancer patients undergoing neoadjuvant anthracycline-based chemotherapy were taken before treatment and 24 h after the first chemotherapy course [57]. The absence of RAD51 foci in treated samples was taken as a sign of HRD. As RAD51 foci formation is confined to cells in the S and G2 phases of the cell cycle, geminin was used as a marker to identify this cell population. The advantage of this approach is that the absence of RAD51 foci in geminin-positive tumor cells actually reflects an inability to perform HR and is not due to the absence of proliferation in the biopsy. Overall, 26% (15/57) of the breast tumor samples showed a low RAD51 score 24 h after anthracycline-based chemotherapy. Compared to a high RAD51 score, the tumors with low RAD51 score post chemotherapy showed a significantly higher percentage of pathological complete response after anthracycline-based neoadjuvant chemotherapy (i.e., 33% vs. 3%, *p =* 0.001).

In another study, tumor biopsies were obtained from 60 primary breast cancer patients 24 h after treatment with epirubicin and cyclophosphamide (EC) [58]. Tumor samples were immunohistochemically stained for RAD51, γH2AX, conjugated ubiquitin, and BRCA1 foci. The results of these stainings were pooled and converted to a DNA damage response (DDR) score of 0–4. The lowest score (0) was considered a measure of HRD. There was a significant inverse correlation between the DDR score and the mean tumor volume reduction determined by helical computed tomography (CT) after four courses of EC chemotherapy. The absence of RAD51 foci before or after one course of chemotherapy did not correlate with significantly more tumor reduction compared to tumors with RAD51 foci present. Importantly, RAD51 foci staining was not corrected for geminin staining, suggesting that this group contains false negative tumors.

### 8.2. RAD51 Foci after Ex Vivo Treatment

An important further improvement of functional testing is the induction of DNA double strand breaks before starting treatment of patients, in order to prevent exposure to therapies that may not provide any benefit. Most commonly, fresh tumor tissue is collected and DNA breaks are induced ex vivo by ionizing radiation (IR). Subsequently, the tumor tissue is incubated for a few hours to allow accumulation of RAD51 protein at the breaks.

A pilot study assessed the feasibility to measure the formation of BRCA1, FANCD2 and RAD51 foci after IR in small pieces of breast tumors ex vivo [59]. RAD51 foci in biopsies indeed could be scored, although quantification was more difficult than in cell lines. A follow up study used the same method in 56 fresh breast cancer patient samples. Ki67 was added as a marker for proliferation in this study, to ensure tumor cells were not quiescent [60]. In total, 22% of the tumors were scored as HRD. Nine HRD and 15 HRP tumors were further characterized using genomic scars and mutational signatures in order to compare this method to other HRD biomarkers. As expected, HRD tumors defined by the RAD51 foci test had a significantly higher level of *BRCA1/2* scars and scored higher on LOH, LST and insertions and deletions than HRP tumors.

As a further improvement, geminin was added to identify S/G2 cells [61] RAD51 foci formation was first investigated in PDX tumors of known *BRCA* status. Tumors with *BRCA1* and *BRCA2* mutations as well as promoter methylation all showed that less than 20% of geminin-positive cells had RAD51 foci, whereas *BRCA* wild type tumors showed foci in more than 50% of the S/G2 cells. This repair capacity (RECAP) test found that 19% of the unselected primary breast tumors (out of 125 breast cancer cases) showed a HRD phenotype, which could be explained by *BRCA1/2* mutations or BRCA1 promoter hypermethylation in most cases [62,63]. However, approximately one third of these HRD tumors did not show clear genetic or epigenetic defects in these genes, suggesting that more than only *BRCA*-deficient tumors may benefit from DSB-inducing therapies, such as PARPi.

A subsequent study on biopsies from metastatic breast cancer patients (*n* = 44) showed that the RECAP test was also feasible in this setting. Importantly, 30% of these biopsies showed HRD, suggesting that an even higher percentage of patients might benefit from PARP inhibitor treatment in metastasized breast cancer. Interestingly, three patients in the HRD group, all having a *BRCA* defect, donated a biopsy before the start of treatment and after progression with DNA double strand break-inducing chemotherapy. Where pre-treatment biopsies were all HRD, post-treatment biopsies of the same patients showed clear induction of RAD51 foci, indicating that this functional assay indeed measures HR capacity in real time [64]. RECAP scores were obtained in 82% of all cases and were available within one week, which makes this test in principle compatible with clinical practice.

This RECAP assay is also feasible for other cancer types. The RECAP assay could also be applied in tissue slices and ascites-derived cancer cells from ovarian tumors [65]. In this first study, the authors found that 10 out of 39 high grade serous ovarian tumors were HRD, mostly because of *BRCA* gene defects. The exact same test has also been used for endometrial cancer. The tumors which histologically resembled ovarian carcinomas more frequently showed HRD rather than endometrioid subtypes [66]. Ascites-derived tumor cells from several other cancer types have also been used for studying RAD51 foci formation [67,68]. Although cell cycle controls are missing here, the data suggest that HRD can also be found in several other tumor types.

### 8.3. RAD51 Foci in Untreated Tumors

A drawback of these functional RAD51 foci assays is the requirement for fresh biopsies. Therefore, another method to use RAD51 foci as a potential biomarker without prior induction of DNA damage was explored. This approach relies on the presence of endogenous DNA double strand breaks in the tumor at the moment of biopsy collection. RAD51 nuclear foci in untreated tumor samples were studied in two small studies. In a cohort of 23 breast cancer patients without a gBRCA mutation but with high suspected hereditary breast cancer, less than 10% RAD51 foci were detected in 14 of 23 tumors [69]. Eleven of the 14 patients with a low RAD51 tumor had a gPALB2 mutation. In the three other tumors with low RAD51 foci, no BRCA1 nuclear foci could be detected, possibly due to BRCA1 promoter methylation. In the nine tumors with RAD51 foci >10%, BRCA1 foci were always detected. In a second study, RAD51 foci tumor staining was performed in seven patients before starting treatment with a PARPi [70]. Three patients showed primary resistance to the PARPi and had RAD51 foci detectable in the tumor prior to treatment initiation and the other four patients with PARPi sensitivity had low RAD51 foci before PARPi started. These results provide some evidence that RAD51 foci in untreated tumor tissue may be an expression of HRD, but the data are not completely consistent and have been studied in a very small number of patient samples so far.

Functional tests have not been extensively tested yet to link biomarkers to predictive value in clinical outcomes. At this moment, several clinical trials have started using RAD51 foci as potential biomarker for HRD selection. If RAD51 foci formation ex vivo can indeed be linked to the clinical outcome of patients, the possibilities for implementation into the clinical decision making should be explored.

## 9. Conclusions and Future Directions

Although several assays have been developed to identify HRD tumors, it is currently not clear which one is the most promising candidate for implementation in clinical practice. The most important first question is whether a test has sufficient predictive value for patient selection. These data are currently lacking for most tests or test results are suboptimal. The MyChoice test was used in several clinical trials in which HRD groups respond better to therapy than the HRP groups, but the non-*BRCA* HRD group benefits much less from PARPi treatment than the g*BRCA-*mutated patients. Furthermore, the HRP subgroup showed survival benefit on PARPi, indicating that withholding treatment based on this test is debatable. Therefore, the first efforts should be geared towards generating sufficient clinical validation of the various tests. It will be important to determine whether the cut off values for the selection of HRD tumors can be optimized in the various tests. Inclusion of too many false positives would decrease the PFS when compared to g*BRCA* mutated subgroups. This is because HRP patients will be treated with a PARPi. However, this might be better than too many false negatives. In that way, the group of PARPi treatments might decline, but a lot of patients will not receive PARPi treatment even though they would benefit from it.

A problem for most tests is the reversion of the HRD phenotype upon treatment. This can, for instance, be caused by mutations and epigenetic silencing [71]. Sequencing-based tests such as the BRCA1/2 classifier and HRDetect/CHORD determine the total mutation load acquired over the course of tumor evolution, implying that resistance due to prior treatment with DNA double strand break-inducing agents might not show up in these sequencing-based tests. For determination of the HR phenotype, the functional tests may be best in predicting therapy response as a real time indicator of the phenotype. Direct clinical validation of this prediction should be obtained in prospective clinical trials.

In addition to the clinical validation of the tests, several other factors will also contribute to their chances of being implemented in clinical decision making. Important considerations are the type of starting material needed, turnaround time of the assay, and cost. FFPE or frozen specimens are most easily handled as the starting material, while functional assays requiring fresh tumor tissue pose a logistic challenge, especially for peripheral hospitals that would need to transport this fresh material to dedicated test laboratories. Therefore, the spontaneous RAD51 foci assay would be much more practical than the RECAP assay, as it does not require handling fresh tumor tissue. Turnaround time is another important factor: test results should be available within two weeks in order to be included in the decision making process. This is possible for most tests, although it is a challenge for WGS approaches. Dedicated sequencing facilities and bioinformatics pipelines would be required to facilitate this time line. Finally, assay costs are also important to select the preferred test. Panel sequencing or shallow Next Generation Sequencing (NGS), as required for the BRCA1/2 classifier) are relatively inexpensive, while good coverage WGS (required for HRDetect and CHORD) is currently the most expensive option.

It is clear that the first step is the clinical validation of the various tests. Several studies have been carried out or are ongoing, but this did not yet result in a gold standard. An interesting approach for clinical validation and direct comparison of the various tests would be a large multicenter trial, in which patients of a certain cancer type are screened for the HRD phenotype using all potential biomarkers. When scored HRD for at least one of the biomarkers, this patient will be considered HRD. Clinical response to PARPi should be analyzed for all different biomarkers. This multicenter clinical trial might also be used to determine cut off values for which the predictive value is optimal. It will be especially important to carry out the functional assay in the various trials, as it requires fresh biopsy material, which cannot be obtained retrospectively. Together with considerations of starting material, turnaround time, and cost, this should then result in the selection of the most optimal HRD test for patient stratification.

## Figures and Tables

**Table 1:** Clinical studies investigating the predictive value of HRD measured by HR-related gene panels.

Type of Study + Ref.	Patients	Number of Genes in Panel + Definition HRD	Subgroups and Distribution of Mutations	Treatment (Number Treated)	Primary Endpoint and Results
TOPARP A trial phase 2; single arm [12]	Men with metastatic castration-resistant prostate cancer who had disease progression after one or two regimens of chemotherapy	113 gene panel on tumor samples HRD: homozygous deletion or deleterious mutation or both in ≥1 HRR genes	16/49 (33%) HRD: 7× *BRCA2*, 5× *ATM*, 3× *FANCA*, 2× *CHEK2*, all others genes 1×	Olaparib 400 mg bid (*n* = 50)	PE: RR defined as CR/PR (RECIST 1.1) or 50% decrease in PSA or CTC count < 5CTCs/7.5 mL Overall RR 16/49 (33%) RR in HRD: 14/16 (88%) RR in HRD excluding *BRCA1/2*: 7/9 (78%) RR in *ATMm* only 4/5 (80%) RR in *FANC2/3m* only (67%) RR non-HRD: 2/33 (6%)
TOPARP-B trial Randomized phase 2 [13]	Men with metastatic castration-resistant prostate cancer who have been treated with one or two taxane regimens	113 gene panel on tumor samples HRD: aberrations in ≥1 HRR genes	161/592 (27%) HRD: 44× *BRCA2*; 40× *ATM*, 33× *CDK12* Of all 161 HRD patients, 98 participated in the study of whom: 32× *BRCA1/2*, 21× *ATM*; 20× *CDK12*; 7× *PALB2*; 18× other	Olaparib 400 mg bid (*n* = 49) vs. olaparib 300 mg bid (*n* = 49)	PE: CCR defined as CR/PR (RECIST 1.1) or 50% decrease in PSA or CTC count < 5CTCs/7.5 mL In both (300 + 400 mg) cohorts together: CCR *BRCA1/2m*: 83.3% (25/30); *PFS 8.3 months* CCR non-*BRCA* HRD group: 30.3% (20/66) CCR *PALB2m* only: 57.1% (4/7); *PFS 5.3 months* CCR *ATMm* only: 36.8% (7/19); *PFS 5.8 months* CCR *CDK12m* only; 25% (5/20); *PFS 2.8 months* CCR of other gene mutations: 20% (2/10)
PROFOUND study Randomized phase 3 trial [14]	Men with metastatic castration-resistant prostate cancer who had disease progression during enzalutamide or abiraterone treatment	15 genes panel on tumor samples HRD: suspected deleterious alterations in at least 1 gene	Cohort A: at least one alteration in *BRCA1/2*, or *ATM* Cohort B: at least one alteration in any of the other 12 genes Main gene mutations: 160× *BRCA1/2*; 92× *ATM*; 94× *CDK12;* 12× *CHEK*2; 8× *PALB2*.	Olaparib vs. nzalutamide or abiraterone (2:1)	PE: iPFS, median FU between 5.4–7.5 months Cohort A (*n* = 256) iPFS 7.4 vs. 3.6 months; HR 0.34 (0.25–0.47) Cohort B (*n* = 142) * estimated iPFS 2.8 vs. 3.2 months iPFS *ATMm* only (*n* = 86): 5.4 vs. 4.7 months; HR 1.04 (0.61–1.87) iPFS *CDK12*m only (*n* = 89): 5.1 vs. 2.2 months; HR 0.74 (0.44–1.31).
CBCSG006 trial Randomized phase 3; post-hoc biomarker analyses [15]	Advanced TNBC, first line, no genomic preselection	28 genes panel in blood samples. HRD defined as at least one gHRR gene mutations	Two groups I: HRD (incl. *gBRCA1/2*; *n* = 63) II: no HRD (*n* = 69) Main gene mutations: 14× *BRCA1/2*, 10× *BARD1,* 9× *ATM*, 6× *BRIP1*, 6× *RAD51C/D,* 26× *FANC*, 5× *PALB2*, *CDH1*, *MSH2/6,* 3× *CHEK2*	Paclitaxel/gemcitabine (GP, *n* = 68) vs. cisplatin/gemcitabine (GT, *n* = 64)	PE: PFS; median FU 54.7 months Group I HRD (incl. *gBRCA1/2m*): PFS: GP vs. GT 10.4 vs. 4.3 months (*p =* 0.011) HRD (excl. g*BRCA1/2m*): * estimated PFS: GP vs. GT: 10.9 vs. 4.7 months Group II; no HRD: PFS GP vs. GT: 6.0 vs. 7.1 months (*p =* 0.154)
TBCRC 048 phase 2, single arm [16]	Metastatic breast cancer, maximum of 2 lines of chemotherapy for advanced disease, no prior PARPi or progression on platinum, and a mutation ≥1 g/sHRRm	20 genes panel on tumor samples and in blood HRD: mutation in ≥1 s/g HRR genes	Cohort 1: Germline mutation in gHRRm genes (excl. g*BRCA1/2m*); *n* = 27 Cohort 2: Somatic mutations in HRR genes (incl. s*BRCA1/2m*); *n* = 27	Olaparib 300 mg bid (*n* = 54)	PE: ORR = PR or CR according to RECIST v1.1 Cohort 1 ORR (gHRRm, excl. *gBRCA1/2m*) 33% (9/27); all 9 had g*PALB2m* ORR in g*PALB2m* only: 82% (9/11) Cohort 2 ORR (sHRRm; incl. *sBRCA1/2m*): 31% (8/26) ORR (sHRRm, excl. s*BRCA*1/2m): 0% (0/10)
Pretermac study Neoadjuvant study, single arm, post-hoc biomarker analyses in TNBC only [17]	Stage II-II primary breast cancer, subset TNBC only	360 genes panel on tumor samples among which HRR genes. HRD: not specifically defined	Three subgroups I all TNBC excl. *gBRCA1/2m* and g*PALB2m*II g/s*BRCAm* and g*PALBm2* only III sHRRm (excl. g/s*BRCAm)* Main gene mutations 6× g/s *BRCA1/2,* *1× gPALB2*; 5× non-*BRCA* sHRR gene mutation (16%): all 1× ATR, EMSY, MEN1, SETD2, PTEN	Olaparib 300 mg bid for up to 10 weeks (*n* = 32)	PE: ORR = PR or CR according to RECIST1.1 ORR all TNBC: 56.3% (18/32) I ORR all TNBC excl. *gBRCA1/2m* and g*PALB2m*: 51.9% (14/27) II: ORR g/s*BRCAm* and g*PALBm2* only: 83% (5/6) III: ORR non-*BRCA* sHRRm: 100% (5/5)
Study 19 Randomized phase 2; post-hoc biomarker analyses [18]	Platinum sensitive grade 2/3 serous ovarian carcinoma, platinum sensitive, 2–3 lines of platinum and an objective response on last platinum-based therapy	287 cancer related genes + select introns from 27 HRR genes + *gBRCA1/2* testing HRD: not specifically defined	Four subgroups (*n* = 209) I: g/s *BRCA*m (*n* = 111); II: g/s*BRCA*wt + sHRRm (*n* = 21); III: g/s*BRCA*wt + sHRR mutation unknown (*n* = 16) IV: g/s*BRCA*wt en sHRRwt (*n* = 58) 209/265 (79%)	Olaparib 400 mg bid vs. placebo as maintenance therapy	PE: PFS olaparib versus placebo PFS in subgroups I: HR 0.16 (0.08–0.30) II: HR 0.21 (0.04–0.86) III: not mentioned IV: HR 0.71 (0.37–1.35)
Prospective cohort study; post hoc biomarker analyses in subgroup of primary tumors only [19]	Ovarian, fallopian tube, or primary peritoneal carcinoma (primary (*n* = 304), recurrent (*n* = 34), paired primary and recurrent (*n* = 24)	13 gene panel in blood and tumor samples. HRD: a deleterious germline and/or somatic mutation in at least one HRR gene	No subgroups Main gene mutations 68× (55%) *BRCA1*; 23× (19%) *BRCA2*; 32× (26%) in the other HRR genes: ATM, BARD1, BRIP1, CHEK1/2, FAM175A, MRE11A, NBN, PALB2, RAD51C/D.	Adjuvant 6× carboplatin/paclitaxel treatment (*n* = 304)	PE: platinum sensitivity (PS) defined as complete response (CR) during adjuvant chemotherapy maintenance of CR >6 months post completion chemotherapy. Analysis in 243 patients with sufficient clinical response data PPS in *gBRCA1/2m*: 81% (38/47) PPS in g/sHRRm (excl. *gBRCA1/2m,* incl. *sBRCA1/2m*) 87% (33/38) PPS in g/sHRRm (excl. *g/sBRCA1/2m*): 78% (14/18) PPS in non-HRD: PPS 60% (95/158)

Bid = twice daily; CCR = confirmed composed response; FU = follow-up; g = germline; HRD homologous recombination deficiency; HR = homologous recombination related; iPFS = imaging-based progression-free survival; ORR = objective response rate; PE = primary endpoint; PFS = progression free survival; s = somatic; TNBC = triple negative breast cancer; *wt* = wild type; * estimated only in case this was possible on the basis of existing data.

**Table 2:** Clinical studies investigating the predictive value of HRD measured by loss of heterozygosity (LOH) or chromosomal aberrations.

**Type of Study + ref.**	**Patients**	**Test Used + Definition HRD**	**Subgroups**	**Treatment** **(Total Treated)**	**Primary Endpoint and Results**
**LOSS OF HETEROZYGOSITY (LOH)**					
ARIEL2 study, phase 2, single arm [20]	Recurrent, platinum sensitive (minimal 1 line platinum-based CTx and PFI >6 months after last platinum dose), high grade serous or endometrioid ovarian, fallopian, or primary peritoneal carcinoma	LOH by Next Generation Sequencing (NGS) HRD = LOH high, i.e., genomic LOH ≥14% on archival or pretreatment biopsies	Three groups I: g/s *BRCA1/2*m (*n* = 40); II: g/s*BRCA1/2*wt + LOH high (*n* = 82); III: g/s*BRCA1/2*wt + LOH low (*n* = 70)	Rucaparib (*n* = 192)	PE: PFS I vs. II vs. III: PFS 12.8 vs. 5.7 vs. 5.2 months I vs. III HR PFS 0.27 (0.16–0.44) II vs. III HR PFS 0.63 (0.42–0.90)
ARIEL3 Randomized phase 3 [21]	Recurrent, platinum sensitive (minimal 2 line platinum-based therapy and PFI >6 months after last platinum dose), high grade serous or endometrioid ovarian, fallopian, or primary peritoneal carcinoma	LOH by NGS HRD = LOH high, i.e., genomic LOH score ≥16% on archival or pretreatment biopsies	Three subgroups I: g/s *BRCAm* (*n* = 196); II: *BRCA*wt + LOH high (*n* = 158); III: *BRCA*wt + LOH low (*n* = 210)	Rucaparib (*n* = 375) vs. placebo (*n* = 189)	PE: iaPFS rucaparib vs. placebo I: iaPFS; 16.6 vs. 5.4 months; HR 0.23 (0.16–0.34) II: iaPFS: 9.7 vs. 5.4 months; HR 0.44 (0.29–0.66) III: iaPFS: 6.7 vs. 5.4 months; HR 0.58 (0.40–0.85)
PrECOG trial Neoadjuvant, Phase 2, single arm [22]	Primary triple negative breast cancer	LOH DNA copy number was determined using genome-wide SNP data HRD = LOH-high, i.e., LOH score of ≥10% in pre-treatment breast biopsies	Two groups I: HRD: LOH high (*n* = 50) II: no-HRD: LOH low (*n* = 15)	6× carboplatin/gemcitabine/iniparib	PE: RCB = residual cancer burden 0 or 1 I: RCB 0/1: 66% II: RCB0/1: 20%
**CHROMOSOMAL ABERRATIONS**					
**Type of study +** **ref.**	**Patients**	**Test used + definition HRD**	**Subgroups**	**Treatment** **(total treated)**	**primary endpoint and results**
VELIA study Randomized Phase 3, first line [23]	High grade serous epithelial ovarian, fallopian tube or primary peritoneal carcinoma; No preselection on platinum sensitivity	MyChoice assay = sum of LOH, TAI and LTS score; HRD = total score ≥42 Second definition HRD = total score ≥33	Three groups I: g/s*BRCA* mutated (298) II: *BRCA*wt + HRD high (329) III: *BRCA*wt with HRD low (372) Of note: 47% HRD within *BRCA*wt	CTx + PB + PB maintance (*n* = 375) vs. CTx + veliparib + PB maintance (*n* = 383) vs. CTx + veliparib + veliparib maintance (*n* = 382)	PE: ia PFS, median FU 28 months I: PFS 34.7 vs. 22.0 months; HR 0.44 (0.28–0.68) II: * estimated PFS 21.5 vs. 18.5 months; HR 0.80 (0.64–1.00) III: PFS 15.0 vs. 11.5 months; HR 0.81 (0.60–1.09)
PRIMA study Randomized phase 3, maintenance therapy after first line therapy [24]	Platinum sensitive high grade serous or endometrioid ovarian carcinoma, stage III or IV, platinum sensitive (i.e., partial or complete response after first line platinum-based chemotherapy)	MyChoice assay = sum of LOH, TAI, and LTS score; *sBRCA1/2m* based on NGS HRD = total score ≥42	Three groups I: g/s*BRCA*m (*n* = 223); II: g/s*BRCA*wt + HRD high (*n* = 150); III: g/s*BRCA*wt + HRD low (*n* = 360) Of note: 29% HRD within *BRCA*wt	Maintenance therapy for 36 months Niraparib (*n* = 484) vs. PB (*n* = 244)	PE: PFS, median FU 13.8 months I: PFS 22.1 vs. 10.9 months; HR 0.40 (0.27–0.62) II: PFS 19.6 vs. 8.2 months; HR 0.50 (0.31–0.83) III: * estimated PFS 6.1 vs. 6.4 months; HR 0.68 (0.49–0.94)
SCOTROC4 study (*n* = 964); randomized phase 3; first line; a prespecified subgroup analyses [25]	Untreated stage III/IV epithelial ovarian, fallopian tube, or primary peritoneal carcinoma; first line	Genome-wide SNP data; sum of LOH, TAI, and LTS score HRD = total score ≥42 Second definition HRD = total score ≥33	Three subgroups I: HRD (incl. s*BRCAm*) (*n* = 79) II: s*BRCA*m only (*n* = 47) III: CCNE1 amplified	Carboplatin/ paclitaxel standard vs. dose intensified based on nadir platelet and neutrophil counts	One of the three PE: PFS (*n* = 225), median FU not mentioned I: PFS 16.5 vs. 9.5 months; HR 0.50 (0.34–0.73) If HRD is defined as ≥33*:* HR 0.51 (0.36–0.72) II: PFS 18.9 vs. 11.0 months; HR 0.48 (0.29–0.79) III: PFS 9.5 vs. 13.2 months; HR 1.56 (1.04–2.34)
PAOLA 1 study, randomized phase 3, second line [26]	High grade serous or endometrioid ovarian, fallopian, or primary peritoneal carcinoma independent of BRCA status *or* non-mucinous epithelial ovarian cancer with a deleterious g*BRCA*m; platinum sensitive after first line carboplatin/taxane bevacizumab	MyChoice assay = sum of LOH, TAI, and LTS score. HRD = total score ≥44	Three subgroups I: g/s*BRCA*m (*n* = 237) II: *BRCA*wt + HRD high (*n* = 152) III: *BRCA*wt + HRD low/unknown (*n* = 419) Of note: 27% HRD within *BRCA*wt	Olaparib + bevacizumab (*n* = 537) vs. PB + bevacizumab (*n* = 269) for a maximum of 24 months	PE: iaPFS, median FU 22.7 months I: iaPFS 37.2 vs. 21.7 months; HR 0.31 (0.20–0.47) II: iaPFS 28.1 vs. 16.6 months; HR 0.43 (0.28–0.66) III: iaPFS 16.9 vs. 16.0 months; HR 0.92 (0.72–1.17)
NOVA trial, randomized phase 3 [27]	High grade serous epithelial ovarian, fallopian tube or primary peritoneal carcinoma; platinum-sensitive (PR or CR and ≥6 months PFI of last platinum containing regimen), ≥2 line of CTx	MyChoice assay = sum of LOH, TAI, and LTS score. HR = total score ≥42	Three subgroups I: g*BRCA*m (*n* = 201) II g/s*BRCA*wt + HRD high (*n* = 115) III g/s*BRCA*wt with HRD low (*n* = 134) Of note: 46% HRD within *BRCA*wt	Niraparib (*n* = 372) vs. PB (*n* = 181) Continue until PD or unacceptable toxicity	PE: PFS, median FU 16.9 months I: PFS 21.0 vs. 5.5 months; HR 0.27 (0.17–0.41) II: *estimated PFS 9.0 vs. 4.0 months; HR 0.38 (0.23–0.63) III: * estimated PFS 5.8 vs. 3.9 months; HR 0.58 (0.36–0.92)
Study 19 Randomized phase 2 study [18]	Platinum sensitive grade 2/3 serous ovarian carcinoma, platinum sensitive (2–3 lines of platinum and objective response on last platinum-based therapy) maintenance therapy.	MyChoice assay = sum of LOH, TAI and LTS score. HRD = total score ≥42 Second definition HRD = total score ≥33	Three subgroups I: g/s *BRCA*m (*n* = 106); II: *BRCA*wt + HRD (*n* = 139); III: *BRCA*wt + no HRD (*n* = 60) Of note: 70% HRD within *BRCA*wt	Olaparib vs. PB *n* = 265 in total; continue until PD or unacceptable toxicity	PE: PFS, exact median FU not mentioned I: PFS in months not mentioned; HR 0.18 (0.10–0.31) II: PFS in months not mentioned; HR 0.48 (0.18–1.27) III: PFS in months not mentioned; HR 0.60 (0.31–1.17) Of note: In subgroup with >6 y PFS, no specific molecular aberrations other than high percentage of g/s*BRCA2*m
Quadra study Phase 2, single arm [28]	High grade serous (grade 2 or 3) epithelial ovarian, fallopian tube or primary peritoneal carcinoma; ≥3 lines of CTx, measurable disease, first line platinum-based CTx PFI must be ≥6 months	MyChoice assay = sum of LOH, TAI, and LTS score. HRD = total score ≥42	Three subgroups I: g*/sBRCA*m (*n* = 87) II g/*sBRCAwt* + HRD (*n* = 135) III g/s*BRCA*wt + no HRD (*n* = 195) Of note: 41% HRD within *BRCA*wt	Niraparib (*n* = 463) until PD or unacceptable toxicity	PE: ia-ORR; median FU 12.2 months I: ia-ORR 29% (39 vs. 27%) II: ia-ORR 15% (26 vs. 10%) III: ia-ORR 3% (3 vs. 4%) secondary outcome: median OS: I: median OS 26.0 (18.1-NR) months II: estimated median OS 14.5 months III: median OS 15.5 (11.6–19.0) months
TBCRC 030 trial neoadjuvant, randomized phase 2 [29]	Stage II-III, TNBC, patients with known *gBRCA1/2m* were excluded	MyChoice assay = sum of LOH, TAI, and LTS score. *BRCA* testing on blood and tumor HRD = total score ≥33 or *g/sBRCA1/2*m	Two subgroups I: HRD (incl g/s*BRCA*m) (*n* = 74) II: no HRD (*n* = 30)	4× Cisplatin (*n* = 56) vs. 12× paclitaxel (*n* = 48)	Two PE: 1st RCB0/1; 2nd pCR I: RCB0/1: 23% vs. 12%; OR 2.22 II: RCB0/1: 29% vs. 31%%; OR 0.90 Of note, 6 (of the 7) *sBRCA*m tumors were randomly allocated to CDDP with only 1 RCB 0/1 (17%) I: pCR: 13% vs. 6%; OR 2.32 II: pCR: 14% vs. 23%%; OR 0.55
GEPARSIXTO trial, randomized phase 3, post hoc analyses in TNBC only [30]	TNBC, stage II-III	MyChoice assay = sum of LOH, TAI, and LTS score. HRD = total score ≥42 or *gBRCA1/2*m	Two subgroups I: HRD or *g/sBRCAm* (*n* = 136) II; no HRD and *BRCAwt* (*n* = 57)	Paclitaxel/ pegylated doxorubicine/ bevacicumab (*n* = 157) vs. paclitaxel/ pegylated doxorubicine/ bevacicumab/carboplatin (*n* = 158)	PE: pCR (ypT0N0) I: pCR 33.9% vs. 63.5%; OR 3.4 (1.7–6.9) II: pCR 20.0% vs. 29.6%; OR 1.7 (0.5–5.7)
TNT Trial. Randomized phase 3, first line [31]	TNBC advanced setting, no CTx for advanced setting	MyChoice assay = sum of LOH, TAI, and LTS score. HRD = total score ≥42	Two subgroups I: g*BRCA*m (*n* = 43) II: HRD (independent of g*BRCA* status) (*n* = 81) III: HRD (incl g*BRCA*m) (*n* = 86)	Carboplatin (*n* = 188) ns Docetaxel (*n* = 188)	PE ORR difference between carboplatin vs. docetaxel per group I: ORR difference: 34.6% vs. −6.4% *p* interaction 0.01 II: ORR difference: −2.2% vs. 2.2% *p* interaction 0.75 III: ORR difference: 5.1% vs. −1.8% *p* interaction 0.63
TBCRC009 study, phase 2, single arm, posthoc analysis [32]	TNBC, maximum of one line CTx in advanced setting	LOH by NGS assay; chromosomal breaks by another assay Sum scores of the two assays HRD-LOH and HRD-LST was used as continues variable; no HRD definition.	Posthoc analysis of difference between responder (CR and PR) vs. non-responders (SD or PD)	carboplatin (*n* = 43) and cisplatin (*n* = 43)	PE ORR Sum score HRD-LOH and HRD-LST was statistically significantly higher among responder than non-responders g*BRCA*wt TNBC patients (12.7 vs. 5.1; *p =* 0.032) No data on ORR for HRD-LOH or HRD-LST scores separately
SWOG S9313 study randomized phase 3, post hoc analyses in TNBC [33]	High risk N0, or low risk *n*+ primary breast cancer, adjuvant chemotherapy.	MyChoice assay = sum of LOH, TAI, and LTS score. HRD = total score ≥42 or *sBRCA1/2*m	Two subgroups I: HRD (incl. *sBRCA1/2*m) II: HRD (all s*BRCA1/2*wt)	Doxorubicine/ Cyclophosphamide concomitant vs. sequential	PE: (5-y) DFS, exact median FU not mentioned I: 5-y DFS: 65.2% vs. 78.7%; HR 0.72 (0.51–1.00) II: 5-y DFS: 65.2% vs. 80.5%; HR 0.64 (0.43–0.94)

CTx = chemotherapy; DFS = disease free survival; FU = follow-up; g = germline; HRD homologous recombination deficiency; HRR = homologous recombination related; iaPFS = investigated assessed PFS; LOH loss of heterozygosity; LTS = large scale state transition score; ORR = objective response rate; OS = overall survival; PE = primary endpoint; PFS = progression free survival; PB = placebo; RCB = residual cancer burden; s = somatic; TAI = telomeric allelic imbalance; TNBC = Triple negative breast cancer; *wt* = wild type; * estimated only in case this was possible on the basis of existing data.

## Data Availability

Not applicable.
